# Provincial variation in colorectal cancer screening adherence in Canada; evidence from the Canadian Partnership for Tomorrow’s Health

**DOI:** 10.3389/fonc.2023.1113907

**Published:** 2023-06-16

**Authors:** Maryam Darvishian, Amina Moustaqim-Barrette, Philip Awadalla, Parveen Bhatti, Philippe Broet, Kelly McDonald, Rachel A. Murphy, Kimberly Skead, Robin Urquhart, Jennifer Vena, Trevor J. B. Dummer

**Affiliations:** ^1^Cancer Control Research, British Columbia (BC) Cancer, Vancouver, BC, Canada; ^2^School of Population and Public Health, University of British Columbia, Vancouver, BC, Canada; ^3^British Columbia (BC) Centre for Disease Control, Vancouver, BC, Canada; ^4^Ontario Institute for Cancer Research, Toronto, ON, Canada; ^5^Department of Molecular Genetics, University of Toronto, Toronto, ON, Canada; ^6^Department of Preventive and Social Medicine, École de Santé Publique, Université de Montréal, Montreal, QC, Canada; ^7^Research Centre, CHU Sainte Justine, Montreal, QC, Canada; ^8^Department of Community Health and Epidemiology, Dalhousie University, Halifax, NS, Canada; ^9^Alberta Health Services, Alberta’s Tomorrow Project, Cancer Research & Analytics, Cancer Care Alberta, Edmonton, AB, Canada

**Keywords:** colorectal cancer, screening, CanPath cohort, adherence, Canada

## Abstract

**Introduction:**

Although colorectal cancer (CRC) screening program is proven to reduce CRC incidence and mortality, understanding patterns and predictors of suboptimal adherence in screening program requires further investigation in Canada.

**Methods:**

We used self-reported data from five regional cohorts of the Canadian Partnership for Tomorrow’s Health (CanPath), namely the BC Generations Project (BCGP), Alberta’s Tomorrow Project (ATP), the Ontario Health Study (OHS), Quebec’s CARTaGENE, and the Atlantic Partnership for Tomorrow’s Health Study (Atlantic PATH). We stratified participants into the following four risk categories: 1) age 50-74 years, 2) family history in a first-degree relative, 3) personal history of chronic inflammatory bowel disease and/or polyps, and 4) co-existence of personal risk and family history. Multivariable logistic regression was used to identify predictors of adherence to the screening guidelines.

**Results:**

Adherence to CRC screening varied considerably between regions, ranging from 16.6% in CARTaGENE to 47.7% in OHS. Compared to the largest cohort OHS, the likelihood of non-adherence to CRC screening was significantly higher in BCGP (OR 1.15, 95% CI 1.11-1.19), the Atlantic PATH (OR 1.90, 95% CI 1.82-1.99) and CARTaGENE (OR 5.10, 95% CI 4.85-5.36). Low physical activity, current smoking, presence of personal risk, family history of CRC significantly reduced the likelihood of adherence to screening recommendations.

**Discussion/conclusion:**

Compared to the national target of ≥ 60% for participation in CRC screening, adherence to regular CRC screening was suboptimal in this cohort of Canadians and varied by region. Further efforts are needed to identify the specific barriers to screening adherence in different provinces and across risk categories.

## Introduction

Colorectal cancer (CRC) is the third most commonly diagnosed cancer in Canada, accounting for 26,900 new cancer cases in 2020 and about half of cases are diagnosed at later stages (1, 2). Screening has proven effective in reducing CRC mortality, through early cancer detection, and in reducing disease incidence, through detection of pre-cancerous lesions (3). According to Canadian guidelines established in 2001 by the Canadian Task Force on Preventive Health Care (CTFPHC), CRC screening is recommended for all individuals aged 50 to 74 years (considered average-risk), and people with a family history of CRC or personal history of ulcerative colitis and/or polyps (considered above-average risk) (4). Briefly, screening recommendations include a fecal occult blood test (FOBT) or fecal immunochemical test (FIT) once every two years, or endoscopy (sigmoidoscopy or colonoscopy) once every 5 years, for average-risk people aged 50-74.

Despite the recommendations, according to data from the 2012 Canadian Community Health Survey, the prevalence of up-to-date self-reported CRC screening tests in Canada in 2012 was 55.2%, ranging from 41.3% in the territories (Northwest Territories, Yukon and Nunavut) to 67.2% in the province of Manitoba (5). A study in Ontario reported only 22% of average-risk participants as ever having been screened and a study in Alberta reported only 23% of average-risk participants as having regular CRC screenings (6, 7) Several characteristics, including younger age, sex, smoking, ethnicity, lack of awareness of guidelines, lower physical activity, and lower educational and income status, showed significant associations with non-adherence to CRC screening (5, 7–10). Provincial differences in the implementation of CRC screening program, as well as having access to primary care clinic/provider or family physician, may partially contribute to the suboptimal adherence to the screening recommendations (11, 12). For example, while the fecal immunochemical test (FIT) is offered annually in Alberta, biennial FIT is recommended in other provinces (2).

Understanding patterns of screening and predictors of screening disparities across Canadian provinces is critical so that barriers to screening can be identified and addressed (2, 7, 12). Although predictors of adherence to CRC screening have been investigated previously, data among individuals with different CRC risk profiles across Canada are limited (11, 13, 14).

To address this gap in knowledge, in this research we used from CanPath—the Canadian Partnership for Tomorrow’s Health (formerly CPTP)—to evaluate regional variation in screening uptake, identify contributing factors to non-adherence to CRC screening, and estimate the adherence to CRC screening among individuals with different risk profiles.

## Methods

### Study population

We utilized data from five of the regional cohorts that contribute to CanPath, including the BC Generations Project (BCGP), Alberta’s Tomorrow Project (ATP), the Ontario Health Study (OHS), Quebec’s CARTaGENE, and the Atlantic Partnership for Tomorrow’s Health Study (Atlantic PATH). At enrollment, participants in each region were asked to complete a Health and Lifestyle Questionnaire (HLQ) that collected data on: age, gender, education, first language, country of birth, race, marital status, income, self-perceived health, presence of comorbid conditions, family history of cancer and chronic diseases, level of physical activity, smoking status and cancer screening history. For the current analysis, the study population was restricted to participants aged 50 to 74 years with no prior diagnosis of CRC. Individuals with missing data on age, cancer status and/or type and CRC screening history were excluded.

### Outcome variable and risk groups

The study outcome was non-adherence to CRC screening. Since CanPath participants were recruited from 2008 to 2016, the 2001 CRC screening guidelines by Canadian Task Force on Preventive Health Care (CTFPHC) were utilized^4^. To examine compliance with national screening guidelines, the HLQ included the following questions on screening status and timing: “have you ever had FOBT?”, “what was the last time you had FOBT?”, “have you ever had endoscopy (i.e., sigmoidoscopy or colonoscopy)?”, and “when was the last time you had a sigmoidoscopy or colonoscopy?” Adherence to screening was then defined as use of FOBT within the past two years and/or endoscopy (i.e., sigmoidoscopy or colonoscopy) within the past 5 years. Based on these data, a binary variable (adherence vs. non-adherence) was created. Study participants were also allocated into one of the following mutually exclusive CRC risk categories: 1) age 50-74 years, without any personal risk of family history of CRC (i.e., average risk), 2) family history in a first-degree relative (i.e., family risk), 3) personal history of chronic inflammatory bowel disease and/or polyps (i.e., personal risk), and 4) personal risk and family history of risk.

### Statistical analysis

Multivariable logistic regression models were used to identify socio-demographic, health, and lifestyle correlates of adherence to screening. Covariates with p-values <0.25 in bivariate regression analysis, or reported to be important as predictors of screening in the scientific literature (age, income, education, province, and marital status), were included in the models. Associations were estimated as odds ratios (ORs) with 95% confidence intervals (95% CI). All models were adjusted for gender, age (i.e., 50-54, 55-59, 60-64, 65-69, and 70-74 years), total annual household income (i.e., < $50,000, $50,000–99,999, ≥$100,000), education (i.e., no education or less than high school, trade, technical school or diploma from a community college, university certificate below bachelor’s level, bachelor’s degree, and graduate degree), marital status (i.e., married or living with a partner, divorced, widowed, separated, single/never married), ethnic background (i.e., white, other), first language (i.e., English, French, other), perception of health (i.e., poor, fair, good, very good, and excellent), country of birth (i.e., Canada, other), smoking status (i.e., never smoked at least 100 cigarettes, past smoker (ever smoked at least 100 cigarettes), current occasional smoker, current daily smoker), and level of physical activity (i.e., low, moderate, or high). Models were also adjusted for presence of comorbidities, defined as any occurrence of at least one of the following conditions: asthma, arthritis or rheumatism, high blood pressure, migraine headaches, chronic bronchitis or emphysema, sinusitis, diabetes, epilepsy, heart disease, cancer, stomach or intestinal ulcers, effects of a stroke, urinary incontinence, bowel disorders, Alzheimer’s disease or dementia, cataracts, glaucoma, and thyroid dysfunction.

Pearson’s chi-square test was used to identify significant difference in non-adherence to screening programs by risk categories (personal risk, family history, personal risk/family history, and average-risk) within each regional cohort. To measure the change in screening rate at guideline-recommended initiation age, the same analysis was conducted among ever-screened individuals (i.e., any lifetime CRC screening (FOBT/endoscopy) aged 50-54 years, who recently became eligible for regular screening. All tests were 2-sided at a significance level of 0.05. All analyses were performed using SAS version 9.4 (Cary, NC, USA). Ethical approval was provided by the Health Research Ethics Board, University of British Columbia.

### Statement of ethics

The study was approved by the Behavioral Research Ethics Board at the University of British Columbia.

## Results

**Figure 1:**
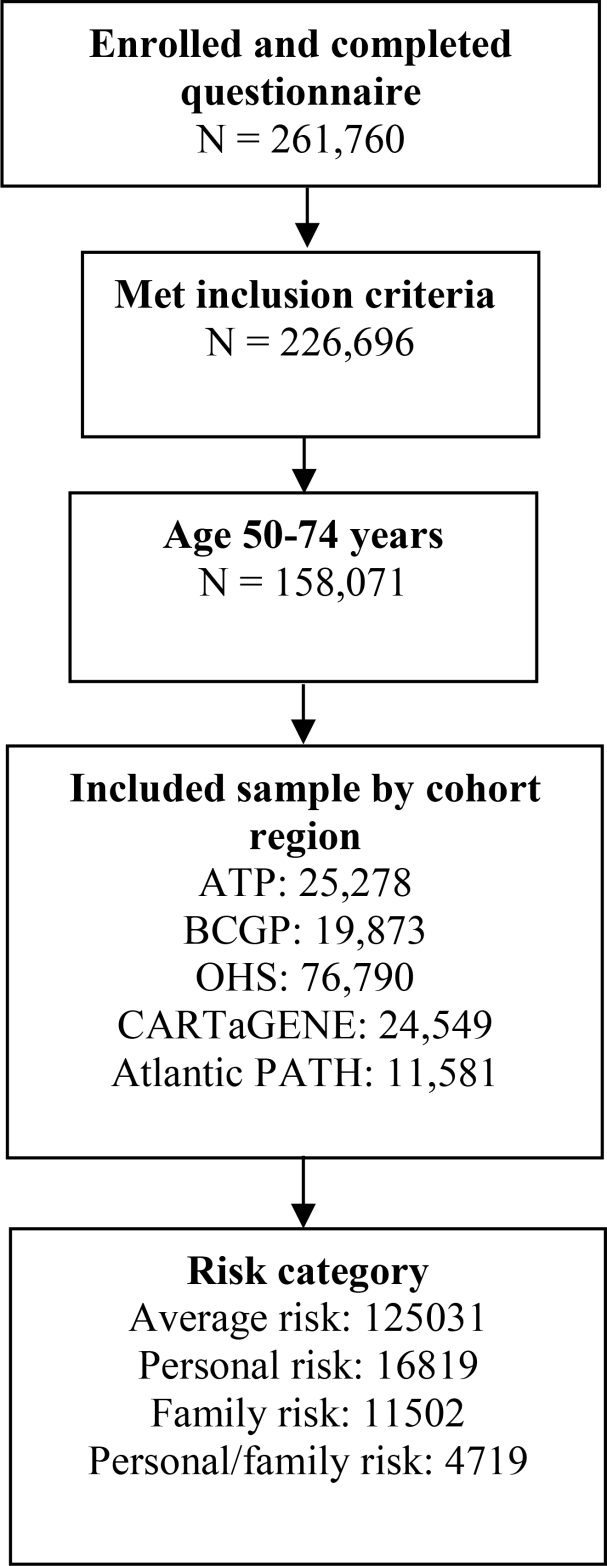
Study Flow diagram.

From a total of 261,760 respondents, 158,071 individuals, including 19,873 (12.6%) from BCGP, 25,278 (16.0%) participants from ATP, 76,790 (48.6%) from OHS, 24,549 (15.5%) from CARTaGENE, and 11,581 (7.3%) from Atlantic PATH, met the inclusion criteria (**Figure 1**). Overall, 125,031 (79.1%) individuals were considered at average risk of developing CRC, 16,819 (10.6%) were considered higher risk due to personal history of ulcerative colitis and/or polyps, 11,502 (7.3%) were also higher risk due to family history of CRC, and 4,719 (3.0%) were considered at highest risk due to both personal history of ulcerative colitis and/or polyps and family history of CRC (**Figure 1**).

Sociodemographic characteristics of study participants are presented in **Table 1**. Overall, a greater proportion of participants were female (58.9%), married or living with a partner (73.5%), white (79.0%), and never smokers (45.8%). Furthermore, greater proportions of participants had household incomes ≥ $100,000 (35.4%), an education level of trade, technical school or diploma from community college (32.4%), very good self-perceived health (40.1%), a high level of physical activity (33.8%), and no comorbid conditions (35.5%).

**Table 1:** Summary Characteristics of Canadian Partnership for Tomorrow’s Health (CanPath), individuals eligible for colorectal cancer screening aged ≥ 50 years, by province (Total N =158, 071).

	Overall (N = 158,071)	Atlantic PATH (N= 11581)	ATP (N=25278)	BCGP (N=19873)	CARTaGENE (N=24549)	OHS (N=76790)
	N (%)	N (%)	N (%)	N (%)	N (%)	N (%)
Lifetime Colorectal Cancer Screening						
No	33012 (20.9)	3078 (26.6)	4119 (16.3)	3803 (19.1)	10886 (44.3)	11126 (14.5)
Yes	125059 (79.1)	8503 (73.4)	21159 (83.7)	16070 (80.9)	13663 (55.7)	65664 (85.5)
Colorectal Cancer Screening Adherence						
No	93093 (58.9)	7834 (67.6)	13625 (53.9)	11020 (55.5)	20469 (83.4)	40145 (52.3)
Yes	64978 (41.1)	3747 (32.4)	11653 (46.1)	8853 (44.5)	4080 (16.6)	36645 (47.7)
Risk Category						
Personal risk	16819 (10.6)	1109 (9.6)	1917 (7.6)	1718 (8.6)	1389 (5.7)	10686 (13.9)
Family risk	11502 (7.3)	1092 (9.4)	2023 (8.0)	1898 (9.6)	1218 (5.0)	5271 (6.9)
Personal risk/family history	4719 (3.0)	515 (4.4)	613 (2.4)	732 (3.7)	110 (0.4)	2749 (3.6)
Average risk	125031 (79.1)	8865 (76.5)	20725 (82.0)	15525 (78.1)	21832 (88.9)	58084 (75.6)
Gender						
Male	64942 (41.1)	3660 (31.6)	9251 (36.6)	6501 (32.7)	11189 (45.6)	34341 (44.7)
Female	93129 (58.9)	7921 (68.4)	16027 (63.4)	13372 (67.3)	13360 (54.4)	42449 (55.3)
Age						
50-54	43460 (27.5)	3396 (29.3)	6884 (27.2)	4090 (20.6)	7982 (32.5)	21108 (27.5)
55-59	42153 (26.7)	3400 (29.4)	6950 (27.5)	4942 (24.9)	6584 (26.8)	20277 (26.4)
60-64	37925 (24.0)	2903 (25.1)	5965 (23.6)	5832 (29.3)	5710 (23.3)	17515 (22.8)
65-69	27910 (17.7)	1736 (15.0)	4388 (17.4)	4986 (25.1)	4166 (17.0)	12634 (16.4)
70-74	6623 (4.2)	146 (1.3)	1091 (4.3)	23 (0.1)	107 (0.4)	5256 (6.8)
Household Income						
< $50,000	14203 (9.0)	944 (8.2)	1784 (7.1)	1228 (6.2)	2219 (9.0)	8028 (10.5)
$50,000 - $99,999	36917 (23.4)	2849 (24.6)	4930 (19.5)	4649 (23.4)	7411 (30.2)	17078 (22.2)
≥ $100,000	55988 (35.4)	4517 (39.0)	8344 (33.0)	7681 (38.7)	8721 (35.5)	26725 (34.8)
Unknown	50963 (32.2)	3271 (28.2)	10220 (40.4)	6315 (31.8)	6198 (25.2)	24959 (32.5)
Education						
No education, or less than high school	37636 (23.8)	2672 (23.1)	6341 (25.1)	4187 (21.1)	6244 (25.4)	18192 (23.7)
Trade, technical school or diploma from community college	51180 (32.4)	4381 (37.8)	9172 (36.3)	6544 (32.9)	7787 (31.7)	23296 (30.3)
University certificate below bachelor’s	8571 (5.4)	649 (5.6)	1273 (5.0)	1229 (6.2)	2039 (8.3)	3381 (4.4)
Bachelor’s degree	36339 (23.0)	2358 (20.4)	5527 (21.9)	4513 (22.7)	5312 (21.6)	18629 (24.3)
Graduate degree	23328 (14.8)	1480 (12.8)	2957 (11.7)	3305 (16.6)	3091 (12.6)	12495 (16.3)
Unknown	1017 (0.6)	41 (0.4)	8 (0.0)	95 (0.5)	76 (0.3)	797 (1.0)
First Language Learned						
English	112984 (71.5)	9941 (85.8)	22141 (87.6)	17388 (87.5)	1732 (7.1)	61782 (80.5)
French	30328 (19.2)	1518 (13.1)	1007 (4.0)	580 (2.9)	21212 (86.4)	6011 (7.8)
Other	14759 (9.3)	122 (1.1)	2130 (8.4)	1905 (9.6)	1605 (6.5)	8997 (11.7)
Marital Status						
Married or living with a partner	116147 (73.5)	9253 (79.9)	19452 (77.0)	14913 (75.0)	16477 (67.1)	56052 (73.0)
Divorced	18030 (11.4)	926 (8.0)	2791 (11.0)	2239 (11.3)	3550 (14.5)	8524 (11.1)
Widowed	7167 (4.5)	526 (4.5)	1279 (5.1)	862 (4.3)	867 (3.5)	3633 (4.7)
Separated	5366 (3.4)	313 (2.7)	507 (2.0)	502 (2.5)	891 (3.6)	3153 (4.1)
Single, never married	10519 (6.7)	523 (4.5)	1246 (4.9)	1267 (6.4)	2721 (11.1)	4762 (6.2)
Unknown	842 (0.5)	40 (0.3)	3 (0.0)	90 (0.5)	43 (0.2)	666 (0.9)
Self-Perceived Health						
Poor	2867 (1.8)	126 (1.1)	179 (0.7)	203 (1.0)	519 (2.1)	1840 (2.4)
Fair	14186 (9.0)	956 (8.3)	1405 (5.6)	1174 (5.9)	2941 (12.0)	7710 (10.0)
Good	52049 (32.9)	3632 (31.4)	7711 (30.5)	5733 (28.8)	10815 (44.1)	24158 (31.5)
Very Good	63312 (40.1)	5065 (43.7)	11582 (45.8)	8616 (43.4)	7746 (31.6)	30303 (39.5)
Excellent	25271 (16.0)	1786 (15.4)	4396 (17.4)	4050 (20.4)	2352 (9.6)	12687 (16.5)
Unknown	386 (0.2)	16 (0.1)	5 (0.0)	97 (0.5)	176 (0.7)	92 (0.1)
Country of Birth						
Canada	128806 (81.5)	11356 (98.1)	21431 (84.8)	15057 (75.8)	22112 (90.1)	58850 (76.6)
Other	29265 (18.5)	225 (1.9)	3847 (15.2)	4816 (24.2)	2437 (9.9)	17940 (23.4)
Ethnicity						
White	124859 (79.0)	9897 (85.5)	15776 (62.4)	17235 (86.7)	17926 (73.0)	64025 (83.4)
Other	33212 (21.0)	1684 (14.5)	9502 (37.6)	2638 (13.3)	6623 (27.0)	12765 (16.6)
Physical Activity Level						
Low	34380 (21.7)	3293 (28.4)	3479 (13.8)	2587 (13.0)	4932 (20.1)	20089 (26.2)
Moderate	43870 (27.8)	3366 (29.1)	5674 (22.4)	4605 (23.2)	7179 (29.2)	23046 (30.0)
High	53404 (33.8)	4354 (37.6)	7984 (31.6)	6828 (34.4)	8705 (35.5)	25533 (33.3)
Unknown	26417 (16.7)	568 (4.9)	8141 (32.2)	5853 (29.5)	3733 (15.2)	8122 (10.6)
Smoking Status						
Never smoked at least 100 cigarettes	72434 (45.8)	5164 (44.6)	12174 (48.2)	9822 (49.4)	9048 (36.9)	36226 (47.2)
Past smoker (ever at least 100 cig)	66071 (41.8)	5162 (44.6)	9143 (36.2)	8919 (44.9)	11387 (46.4)	31460 (41)
Current occasional smoker	3205 (2.0)	238 (2.1)	319 (1.3)	248 (1.2)	885 (3.6)	1515 (2.0)
Current daily smoker	13340 (8.4)	896 (7.7)	1563 (6.2)	705 (3.5)	3137 (12.8)	7039 (9.2)
Unknown	3021 (1.9)	121 (1.0)	2079 (8.2)	179 (0.9)	92 (0.4)	550 (0.7)
Number of comorbidity						
0	56157 (35.5)	4343 (37.5)	9908 (39.2)	7115 (35.8)	8927 (36.4)	25864 (33.7)
1	47454 (30)	4019 (34.7)	8809 (34.8)	5689 (28.6)	4798 (19.5)	24139 (31.4)
2	26627 (16.8)	2201 (19.0)	4623 (18.3)	2946 (14.8)	3061 (12.5)	13796 (18.0)
3	9106 (5.8)	770 (6.6)	1466 (5.8)	909 (4.6)	877 (3.6)	5084 (6.6)
4	4047 (2.6)	188 (1.6)	377 (1.5)	471 (2.4)	1123 (4.6)	1888 (2.5)
5	14567 (9.2)	44 (0.4)	91 (0.4)	2705 (13.6)	5763 (23.5)	5964 (7.8)
Unknown	113 (0.1)	16 (0.1)	4 (0.0)	38 (0.2)	0 (0.0)	55 (0.1)

Among regions, a greater proportion of participants in OHS (13.9%) and BCGP (9.6%) had personal risk and family risk of CRC, respectively (**Table 1**). While the proportion of low level of physical activity was greater among Atlantic PATH participants (28.4%), a higher proportion of participants in CARTaGENE were among current daily smokers (12.8%). Most participants had reported screening for CRC at least once (79.1%), ranging from 55.7% in CARTaGENE to 85.5% in OHS (**Table 1**). However, adherence to CRC screening guidelines was considerably lower (overall: 41.1%), ranging from 16.6% in CARTaGENE to 47.7% in OHS (**Table 1**).

Compared to the largest CanPath region, OHS, the odds of non-adherence to CRC screening were significantly higher in BCGP (OR 1.15, 95% CI 1.11-1.19), Atlantic PATH (OR 1.90, 95% CI 1.82-1.99) and CARTaGENE (OR 5.10, 95% CI 4.85-5.36). Male gender (OR 0.92, 95% CI 0.90-0.94), Canada as country of birth (OR 0.97, 95% CI 0.94-0.99), presence of two or more comorbid conditions (e.g., presence of 5 comorbid conditions: OR 0.87, 95 CI 0.83-0.91), and older age (age 70-74: OR 0.52, 95% CI 0.49-0.55) were significantly associated with lower non-adherence to screening recommendations (**Table 2**). Compared to individuals with high level of physical activity, odds of non-adherence to screening recommendations were significantly higher among individuals with low (OR 1.13, 95% CI 1.10-1.17) and moderate (OR 1.33, 95% CI 1.29-1.38) levels of physical activity. Furthermore, compared to average-risk individuals, individuals with personal risk (OR 1.29, 95% CI 1.25-1.34), family history (OR 1.35, 1.29-1.40), and personal risk and family history (OR 1.61, 95% CI 1.51-1.72) had significantly higher odds of non-adherence to CRC screening. Finally, compared to individuals who never smoked at least 100 cigarettes and individuals with white ethnic background, current daily smokers (OR 1.28, 95% CI 1.23-1.34) and those of other ethnicity (OR 1.05, 1.03-1.09), respectively, had significantly higher non-adherence to CRC screening.

**Table 2:** Predictors of non-adherence to the colorectal cancer screening programs (Total N =158, 071).

Variable	Non-adherence N (%)	Adherence N (%)	Odds Ratio for Non-Adherence * (95%CI)	*P*-Value
Cohort				
BCGP	11020 (11.84)	8853 (13.62)	1.15 (1.11,1.19)	<.0001
Atlantic Path	7834 (8.41)	3747 (5.77)	1.90 (1.82,1.99)	<.0001
ATP	13625 (14.64)	11653 (17.93)	1.01 (0.98,1.04)	0.6353
CARTaGENE	20469 (21.99)	4080 (6.27)	5.10 (4.85,5.36)	<.0001
OHS	40145 (43.12)	36645 (56.39)	1.00	
Risk Category				
Personal risk	9870 (10.60)	6949 (10.69)	1.29 (1.25,1.34)	<.0001
Family risk	7248 (7.79)	4254 (6.55)	1.35 (1.29,1.40)	<.0001
Personal risk/family history	2971 (3.19)	1748 (2.69)	1.61 (1.51,1.72)	<.0001
Average risk	73004 (78.42)	52027 (80.07)	1.00	
Gender				
Female	55830 (59.97)	37299 (57.40)	1.00	
Male	37263 (40.03)	27679 (42.60)	0.92 (0.9,0.94)	<.0001
Age				
50-54	29172 (31.34)	14288 (21.99)	1.00	
55-59	25154 (27.02)	16999 (26.16)	0.75 (0.72,0.77)	<.0001
60-64	21087 (22.65)	16838 (25.91)	0.64 (0.62,0.66)	<.0001
65-69	14749 (15.84)	13161 (20.25)	0.58 (0.56,0.60)	<.0001
70-74	2931 (3.15)	3692 (5.68)	0.52 (0.49,0.55)	<.0001
Household Income				
< $50,000	7976 (8.57)	6227 (9.58)	0.92 (0.89,0.95)	<.0001
$50,000 - $99,999	22169 (23.81)	14748 (22.70)	0.92 (0.90,0.95)	<.0001
≥ $100,000	32552 (34.97)	23436 (36.07)	1.00	
Unknown	30396 (32.65)	20567 (31.65)	0.84 (0.81,0.88)	<.0001
Education				
Unknown	564 (0.61)	453 (0.69)	0.97 (0.85,1.10)	0.6213
No education, or less than high school	22341 (24.00)	15295 (23.53)	0.99 (0.96,1.03)	0.6243
Trade, technical school or diploma from community college	30364 (32.62)	20816 (32.04)	0.99 (0.95,1.02)	0.4404
University certificate below bachelor’s	5293 (5.69)	3278 (5.05)	1.03 (0.98,1.09)	0.2538
Bachelor’s degree	21128 (22.70)	15211 (23.41)	0.98 (0.95,1.01)	0.2319
Graduate degree	13403 (14.39)	9925 (15.27)	1.00	
First Language Learned				
English	62144 (66.75)	50840 (78.24)	1.00	
French	22459 (24.12)	7869 (12.11)	0.88 (0.85,0.92)	<.0001
Other	8490 (9.12)	6269 (9.65)	1.05 (1.01,1.09)	0.0262
Marital Status				
Married or living with a partner	67111 (72.09)	49036 (75.46)	1.00	
Divorced	11156 (11.98)	6874 (10.58)	1.13 (1.09,1.17)	<.0001
Widowed	4103 (4.41)	3064 (4.71)	1.13 (1.07,1.19)	<.0001
Separated	3412 (3.66)	1954 (3.01)	1.20 (1.13,1.27)	<.0001
Single, never married	6844 (7.35)	3675 (5.66)	1.14 (1.09,1.19)	<.0001
Unknown	467 (0.50)	375 (0.58)	1.01 (0.88,1.16)	0.9192
Self-Perceived Health				
Unknown	267 (0.29)	119 (0.18)	1.05 (0.83,1.33)	0.6767
Poor	1729 (1.86)	1138 (1.75)	1.09 (1.00,1.19)	0.0547
Fair	8806 (9.46)	5380 (8.28)	1.16 (1.10,1.22)	<.0001
Good	31795 (34.15)	20254 (31.17)	1.10 (1.07,1.14)	<.0001
Very Good	36213 (38.90)	27099 (41.70)	1.02 (0.99,1.05)	0.2473
Excellent	14283 (15.34)	10988 (16.91)	1.00	
Country of Birth				
Canada	77114 (82.83)	51692 (79.55)	0.97 (0.94,0.99)	0.0413
Other	15979 (17.17)	13286 (20.45)	1.00	
Race/Cultural Origin				
White	72444 (77.82)	52415 (80.67)	1.00	
Other	20649 (22.18)	12563 (19.33)	1.05 (1.03,1.09)	0.0003
Physical Activity Level				
Unknown	16326 (17.54)	10091 (15.54)	1.33 (1.29,1.38)	<.0001
Low	20631 (22.16)	13749 (21.16)	1.13 (1.10,1.17)	<.0001
Moderate	25532 (27.43)	18338 (28.23)	1.04 (1.02,1.07)	0.0016
High	30604 (32.87)	22800 (35.09)	1.00	
Smoking Status				
Never smoked at least 100 cigarettes	41714 (44.81)	30720 (42.28)	1.00	
Past smoker (ever smoked at least 100 cig)	38677 (0.16)	27394 (42.16)	1.01 (0.99,1.03)	0.3661
Current occasional smoker	2059 (0.05)	1146 (1.76)	1.04 (0.96,1.12)	0.3395
Current daily smoker	9011 (9.68)	4329 (6.66)	1.28 (1.23,1.34)	<.0001
Unknown	1632 (1.75)	1389 (2.14)	0.99 (0.92,1.07)	0.8186
Presence of Comorbidity				
Unknown	62 (0.07)	51 (0.08)	1.02 (0.70,1.48)	0.9180
0	34605 (37.17)	21552 (33.17)	1.12 (1.09,1.15)	<.0001
1	26846 (28.84)	20608 (31.71)	1.00	
2	14843 (15.94)	11784 (18.13)	0.95 (0.92,0.98)	0.0018
3	4964 (5.33)	4142 (6.37)	0.90 (0.86,0.95)	<.0001
4	2475 (2.66)	1572 (2.42)	0.92 (0.86,0.99)	0.0304
5	9298 (10.00)	5269 (8.11)	0.87 (0.83,0.91)	<.0001

*Adjusted for all other factors listed in the table.

**Figure 2:**
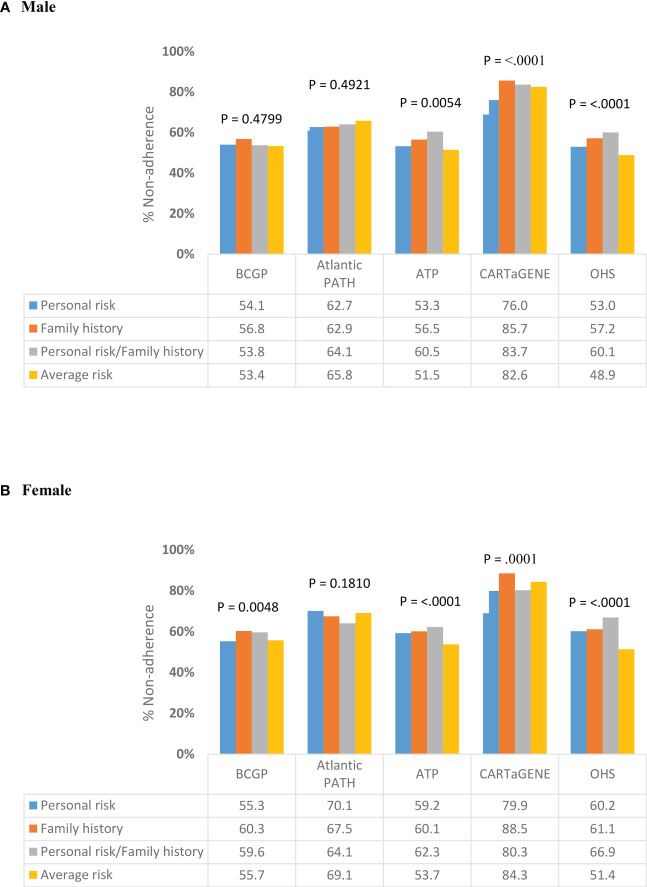
Variation in non-adherence to CRC screening programs by risk category, stratified by **(A)** Male **(B)** Female.

**Figure 2** presents the proportion of screened individuals in each region, stratified by gender. In BCGP, no significant differences in percent non-adherence by risk category was observed among males; however, among females, non-adherence was lowest among those with average (55.7%) and personal risks (55.3%) as compared to those with family history (60.3%) or with both personal risk and family history (59.6%) (**Figures 2A, B** ). In ATP, OHS, and CARTaGENE, significant differences in % non-adherence by risk category were observed among both males and females. In ATP and OHS, the lowest and highest non-adherence was observed among the average risk category and the personal/family history risk category, respectively, among both men and wome (**Figures 2A, B**). In CARTaGENE, among females, the lowest percent non-adherence was observed among those in the personal history (79.9%) and personal risk/family history (80.3%) categories.

**Figure 3:**
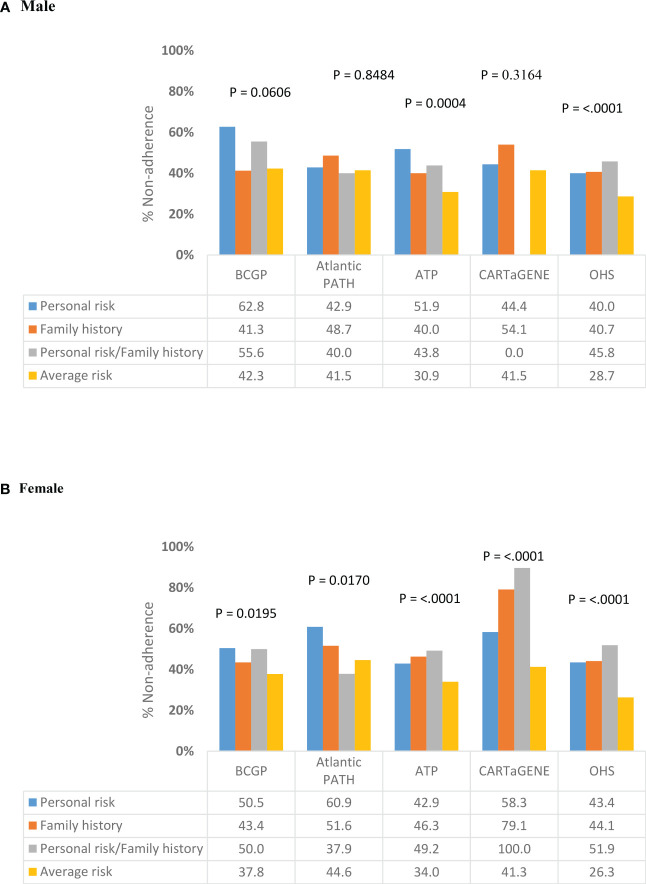
Variation in non-adherence to CRC screening programs by risk category among ever-screened individuals aged 50-54 years, stratified by gender (Total N =20,307). **(A)** Male **(B)** Female.

Among ever-screened individuals aged 50-54, the percent non-adherence was significantly different across risk categories for males in BCGP, ATP and OHS (**Figure 3A**). In BCGP, percent non-adherence was lowest among men in the family history (N = 31; 41.3%) and average risk (N = 190; 42.3%) categories and highest among men in the personal risk category (N= 27; 62.8%). In ATP, percent non-adherence was lowest among men in the average risk category (N = 337; 30.9%) and highest among men in the personal risk category (N = 42; 51.9%). Among females, significant differences in non-adherence were observed in each of the cohorts (**Figure 3B**). Percent non-adherence was lowest among those women with average risk in BCGP (N = 488; 37.8%), ATP (N = 820; 34.0%), OHS (N = 1495; 26.3%) and CARTaGENE (N = 301; 41.3%) and lowest among those with both personal risk and family history in Atlantic PATH (N = 11; 37.9%). In BCGP, percent non-adherence was highest among those with personal risk (N = 53; 50.5%) and with both personal risk and family history (N = 50; 50.0%). Percentage non-adherence was highest among those with both personal risk and family history in ATP (N = 30; 49.2%), OHS (N = 69; 51.9%), and CARTaGENE (N = 1; 100%). In Atlantic PATH, percent non-adherence was highest among those in the personal risk category (N = 53; 60.9%).

**Figure 4:**
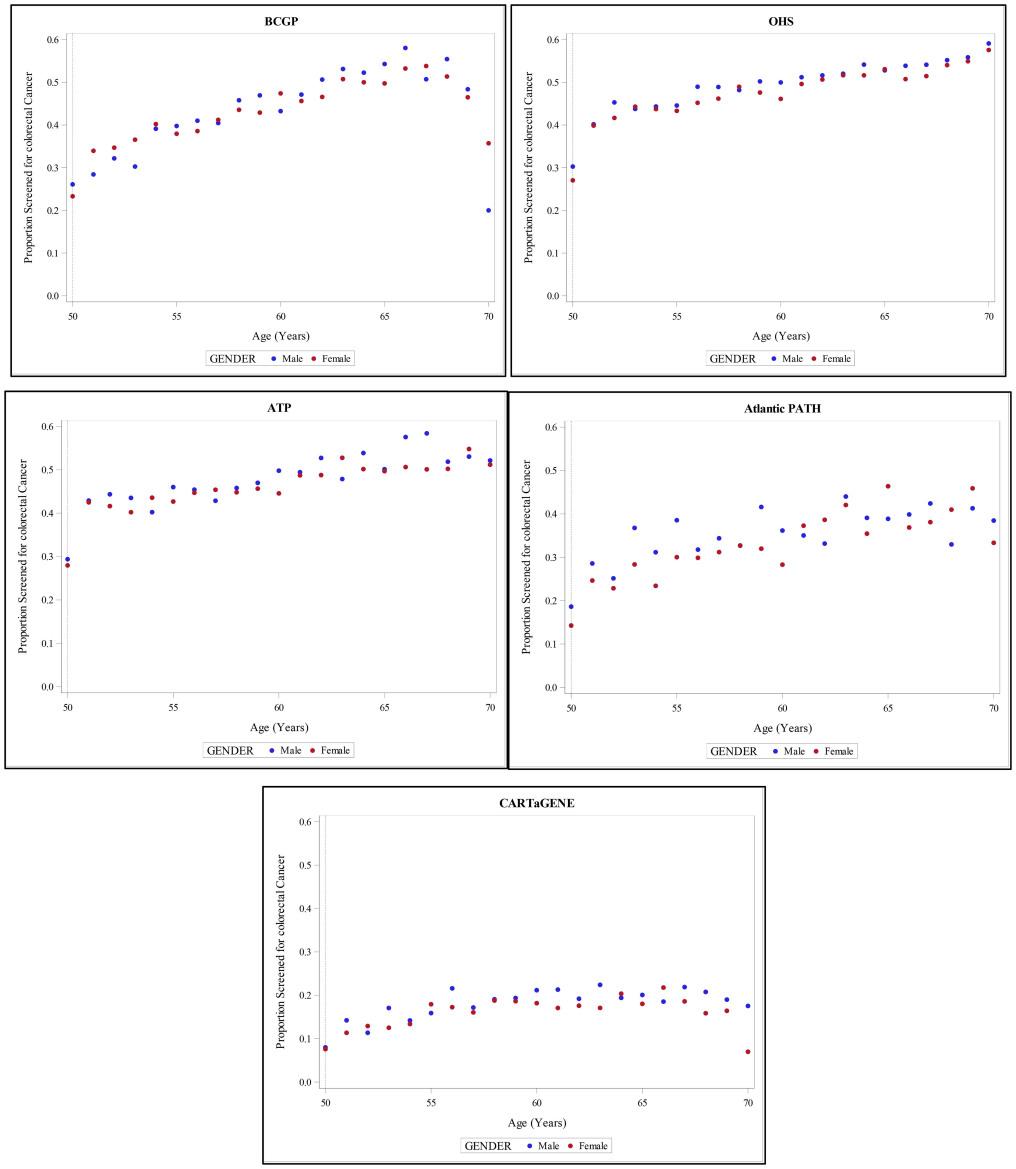
Proportion of screened individuals in each province, stratified by age and gender.

**Figure 4** provides an overall overview of proportion of screened individuals in each province, stratified by age and gender.

## Discussion

We assessed adherence to CRC screening guidelines across five CanPath regional cohorts, taking into account baseline CRC risk profiles. In general, compared to participants in OHS (the largest CanPath regional cohort), the likelihood of non-adherence to CRC screening guidelines was significantly higher among participants in BCGP, Atlantic PATH, and CARTaGENE, but not in ATP. Additionally, the proportion of non-adherence to screening recommendations by risk categories varied significantly within and between provinces.

According to a Canadian Community Health Survey conducted in 2012, the prevalence of CRC screening among individuals 50-74 years was 55.2%, which was higher than the adherence we observed in CanPath (41.1%) (5). Consistent with our findings, in the 2012 survey, the highest screening prevalence was reported in Ontario (64.1%) and the lowest was in Quebec (43.4%) (5).

The observed geographic variability in adherence to CRC screening could be related to level of urbanization, access to care, education and income status (12). Although education and socioeconomic status (SES) were not associated with adherence to screening recommendations in our study, the observed differences among individuals with different ethnic backgrounds and countries of birth might be an indicator for the potential impact of urbanization and income strata on screening compliance (12). For instance, in a study conducted by Simkin et al., higher income, across all levels of urbanization, was associated with increased odds of CRC screening compared to the lowest income quintile (12). In our study, non-adherence decreased with increasing number of comorbid conditions, which could partly highlight the impact of access to care as well as health-care seeking behavior on adherence to screening among individuals with chronic medical conditions.

The few studies that have assessed adherence to CRC screening among individuals with personal risk and/or family history of colorectal cancer reported higher screening uptake (7). In contrast, we found that compared to average-risk individuals, non-adherence to screening programs was significantly higher among those with a personal risk and/or family history of CRC (15). Biases from the use of self-reported questionnaire data may be a potential explanation for these discrepant findings (16). However, a review in 2017 of guidelines for CRC screening in those with a family history of CRC, by the Canadian Association of Gastroenterology, found heterogeneity in recommendations and challenges for family physicians in identifying screening modalities for high-risk individual (17). The review resulted in revised recommendations for CRC screening for at-risk individuals, implemented in 2018 (17). It may be that our findings reflect inconsistencies and heterogeneity in CRC screening for high-risk individuals prior to this time.

According to the CTFPHC in 2016, the screening recommendation is categorized as weak for individuals aged 50 to 59 and strong for individuals aged 60 to 74 (18). While the incidence of CRC is declining among older individuals, it is on the rise among younger adults aged <50 years (2, 19). Hence, the benefits of screening among individuals aged <50 years with personal risk and/or family history of CRC should be carefully assessed by the clinicians (19). Consistent with previous studies, we observed that socio-demographic characteristics of participants, namely older age, male gender, white ethnic background, and married or living with a partner status were associated with lower non-adherence, and smoking and identifying as immigrant were associated with higher non-adherence to the screening guidelines (5, 7, 11, 20).

CanPath participants were recruited from 2008 to 2014, and as such, the 2001 CRC screening guidelines by CTFPHC were applied in this study (4). The 2016 CTFPHC guideline recommended fecal occult blood test (FOBT) or fecal immunochemical test (FIT) once every two years, or endoscopy (sigmoidoscopy or colonoscopy) once every 5 years, for asymptomatic people aged 50-74 (21). The reliability and efficacy of all three of these methods have been well established, with several randomized controlled trials (RCTs) demonstrating 15% to 33% reductions in CRC mortality as a result of screening with FOBT, FIT, and endoscopy (22, 23). Currently, the updated 2016 recommendations of screening asymptomatic individuals, 50-74 years, at average-risk of developing CRC, every 12-30 months with a fecal test (FT) (i.e., guaiac fecal test (FTg) or FIT) is being used (24). Varied implementation of organized population-based CRC screening programs across provinces may potentially impact the observed variation in adherence rates across the provinces and territories in this study. For example, while FIT is recommended in Alberta every year, in Quebec, the CRC screening program is still in a pilot phase, which could explain the lowest adherence rate compared to other provinces (2). Hence, adherence and compliance to the updated recommendations among the various risk groups as well as the potential impact of specific provincial guidelines should be evaluated in a future study.

CanPath includes a large sample drawn from across different regions of Canada (25). To our knowledge, this is the first pan-Canadian study to assess adherence to CRC screening guidelines while considering individual CRC risk profiles across different regions. By using detailed data from Canada’s largest cohort study, we were able to assess the impact of several potential confounding factors, such as cigarette smoking, level of physical activity, and self-perceived health. The standardized data collection questionnaire used in CanPath support the internal validity of the cohort (25). However, the self-reported nature of the screening information may potentially introduce bias (e.g., recall bias) into the adherence estimates reported in this study, although this is unlikely to be differential across the different groups (26, 27). Additionally, the voluntary participation of study participants could potentially limit generalizability of the findings and reduce the external validity (25, 28). However, due to the similar prevalence of common chronic diseases and unhealthy lifestyle behaviors in the CanPath cohort compared to national rates, a “healthy volunteer effect” in CanPath is unlikely to materially impact the findings (25, 29). Finally, although in this study the impact of other potential confounding factors, such as substance use, could not been assessed, we expect that the observed association between current smoking status and self-perceived health status with adherence to screening could be used as a proxy for potential effect of these unmeasured confounders on the outcome (30).

In conclusion, the overall adherence to screening guidelines among average-risk individuals in the CanPath cohort was suboptimal (41.1%) and varied considerably by region. Additionally, adherence within regions varied by personal CRC risk categories. Efforts are needed to further explore barriers to CRC screening adherence across Canada and to assess disparities in access to screening programs among individuals with different risk profiles.

## Data availability statement

The data analyzed in this study is subject to the following licenses/restrictions: “The data that support the findings of this study are available on request from CanPath – The Canadian Partnership for Tomorrow’s Health (formerly CPTP). The data are not publicly available due to privacy or ethical restrictions”. Requests to access these datasets should be directed to CanPath, https://canpath.ca/access-process/.

## Ethics statement

The studies involving human participants were reviewed and approved by Behavioral Research Ethics Board, University of British Columbia. The patients/participants provided their written informed consent to participate in this study.

## Author contributions

PA, PBh, PBr, KM, KS, RU, JV, TD (data collection); MD, AM-B, TD, RM (study conception and design); MD, AM-B (data analysis, drafting initial manuscript); All authors contributed to the article and approved the submitted version.
